# Preparation of Crown Ether-Containing Polyamide Membranes via Interfacial Polymerization and Their Desalination Performance

**DOI:** 10.3390/membranes15030077

**Published:** 2025-03-03

**Authors:** Liqing Xing, Liping Lin, Jiaxin Guo, Xinping He, Chunhai Yi

**Affiliations:** Shaanxi Key Laboratory of Energy Chemical Process Intensification, Department of Chemical Engineering, Xi’an Jiaotong University, Xi’an 710049, China; 15956994@stu.xjtu.edu.cn (L.X.); liping_lin@xjtu.edu.cn (L.L.); jxguo@xjtu.edu.cn (J.G.); xinpinghe@xjtu.edu.cn (X.H.)

**Keywords:** crown ether, interfacial polymerization, RO membrane, polyamide

## Abstract

The large-scale application of aromatic polyamide (PA) thin-film composite (TFC) membranes for reverse osmosis has provided an effective way to address worldwide water scarcity. However, the water permeability and salt rejection capabilities of the PA membrane remain limited. In this work, cyclic micropores based on crown ether were introduced into the PA layer using a layer-by-layer interfacial polymerization (LbL-IP) method. After interfacial polymerization between m-phenylenediamine (MPD) and trimesoyl chloride (TMC), the di(aminobenzo)-18-crown-6 (DAB18C6) solution in methanol was poured on the membrane to react with the residual TMC. The cyclic micropores of DAB18C6 provided the membrane with rapid water transport channels and improved ion rejection due to its hydrophilicity and size sieving effect. The membranes were characterized by FTIR, XPS, SEM, and AFM. Compared to unmodified membranes, the water contact angle decreased from 54.1° to 31.6° indicating better hydrophilicity. Moreover, the crown ether-modified membrane exhibited both higher permeability and enhanced rejection performance. The permeability of the crown ether-modified membrane was more than ten times higher than unmodified membranes with a rejection above 95% for Na_2_SO_4_, MgSO_4_, MgCl_2,_ and NaCl solution. These results highlight the potential of this straightforward surface grafting strategy and the modified membranes for advanced water treatment technologies, particularly in addressing seawater desalination challenges.

## 1. Introduction

The combination of population growth, rapid economic development, and water pollution induced by industrialization, has promoted water scarcity to one of the world’s greatest challenges [1]. It is estimated that by 2030, global water demand will reach 6900 billion cubic meters annually, far exceeding the supply capacity of surface water resources [2,3,4]. Studies indicate that 96.5% of the Earth’s water is seawater, while only 0.8% is freshwater directly available for human consumption [5]. Therefore, seawater desalination has become a critical solution. Current research on desalination methods focuses on two main approaches: thermal processes involving phase changes and innovative membrane-based separation techniques. Among these, reverse osmosis (RO) technology, based on membrane separation, is undoubtedly one of the most environmentally friendly and energy-efficient approaches.

In terms of the reverse osmosis (RO) membrane structure, current research primarily focuses on asymmetric membranes and composite membranes. Since 1972, when Cadotte et al. developed thin-film composite (TFC) membranes for RO processes, TFC RO membranes have been widely used in wastewater reuse, brackish water and seawater desalination, and the production of ultra-pure water [6]. These RO membranes are polyamide (PA) TFC RO membranes fabricated via interfacial polymerization (IP), where the active PA layer ensures selective permeability. Despite their high efficiency, environmental friendliness, and compact design, PA TFC membranes still face several common challenges in practical applications.

In modern polyamide (PA)-based RO membranes, flux is a critical performance parameter because large-scale seawater desalination demands higher flux to improve overall process efficiency. Strategies for fabricating high-permeability membranes typically include reducing the dense layer thickness, incorporating additives, or using inorganic nanoparticles to create organic–inorganic hybrid membranes. For instance, Asempour et al. improved membrane permeability by introducing poly(amidoamine) (PAMAM) dendrimers functionalized halloysite nanotubes (HNTs) [7]. Shi et al. enhanced the membrane’s performance by optimizing the support layer and incorporating hydroxy-rich tannic acid into it during the fabrication process [8]. Yung et al. discovered that hydrophilic additives could create additional water channels in the separation layer. Long-term operational tests confirmed stable membrane performance [9]. Since the beginning of the 21st century, research has increasingly focused on hydrophilic nanomaterials. Nanomaterials have attracted significant attention due to their unique surface functional groups and excellent compatibility with the polymer matrix in membrane fabrication [10].

Traditionally, the pores in reverse osmosis (RO) and nanofiltration (NF) membranes are believed to originate from voids between polymer chains or within polymer aggregates. The pore size and its distribution are determined by crosslinking reactions and the aggregation of polymer chains, resulting in a relatively broad pore size distribution [11]. Developing membranes with appropriate pore sizes and narrower pore size distributions remains a significant challenge for RO and NF membranes.

**Figure 1:**
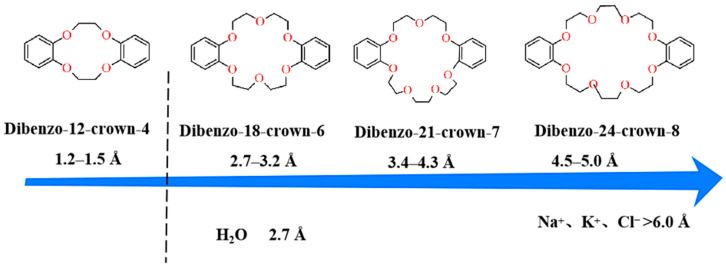
Different crown ether structures and their hydration radii.

The pore size of crown ether compounds depends on the number of ether oxygen atoms in the crown ring, ranging from approximately 1.2 Å to over 4 Å. This study utilizes the cyclic micropores of crown ethers [12,13,14] to create rapid water transport channels and achieve efficient ion separation. The ether bonds in crown ethers exhibit excellent hydrophilicity, while their specific micropore sizes enable the construction of channels within membranes for rapid water transport and efficient ion rejection [15]. As shown in Figure 1, there is a significant size difference between water molecules and hydrated ions such as NO^3−^, Cl^−^, Ga^2+^, K^+^, Na^+^, and Li^+^ [16,17,18]. The diameter of hydrated ions exceeds 6 Å, while that of water molecules is only 2.7 Å.

Crown ether molecules containing cyclic micropores ranged in size from 1.2~5.0 Å with varying oxygen atom numbers [19]. The pore sizes of 18-crown-6 to 24-crown-8 are larger than water molecules but smaller than hydrated ions. Meanwhile, the ether groups in crown ethers display good hydrophilicity. Their size-selective properties and affinity for water molecules enable high permeability and high rejection rates. Therefore, optimal micropore size and excellent hydrophilicity of crown ethers [20,21] make them potential materials for RO membranes. Based on this theory, this study designed and synthesized crown ether derivatives from monomer structures and incorporated them into the fabrication of RO membranes.

In this work, di(aminobenzo)-18-crown-6 (DAB18C6) was synthesized by the nitration and hydrogenation reaction of dibenzo-18-crown-6. The amino groups of DAB18C6 reacted with trimesoyl chloride (TMC) to form a cross-linking network. And due to the poor solubility of DAB18C6 in water, the membrane could not be prepared following the typical IP method. A layer-by-layer interfacial polymerization method [22,23,24] was applied to introduce crown ethers onto the membrane surface. A secondary interfacial polymerization technique was employed, providing a novel approach to the preparation of crown ether-based reverse osmosis membranes. The modified membranes exhibited significantly improved permeability while maintaining high salt rejection rates, which offers a potential opportunity for highly efficient water treatment.

## 2. Materials and Methods

### 2.1. Materials and Chemicals

The polysulfone ultrafiltration membrane, with a cut-off molecular weight of 5000 Da, was purchased from Shandong Jiuzhang Membrane Technology Co., Ltd. (Dongying, China). The chemicals, including meta-phenylenediamine (MPD), trimesoyl chloride (TMC), n-hexane, sodium dodecyl sulfate, anhydrous sodium sulfate, ethylene glycol monomethyl ether, hydrazine hydrate (80% solution), Pd-C catalyst (10 wt% Pd), and N,N-dimethylformamide, were ordered from Beijing J&K Scientific Co., Ltd (Beijing, China).

### 2.2. Preparation of Diamino-Modified Crown Ethers

**Figure 2:**
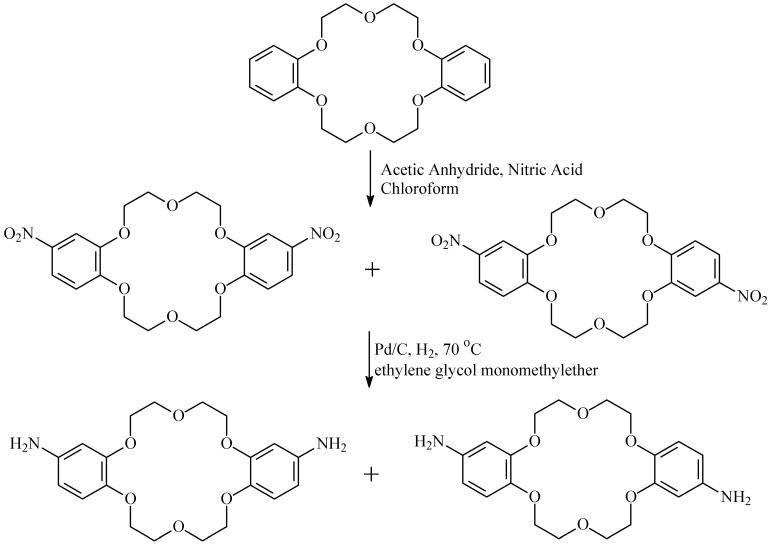
The synthesis of diamino-dibenzo-18-crown-6.

Diamino-dibenzo-18-crown-6 (DAB18C6) was prepared according to our previous work [25]. Firstly, dinitrobenzo-dibenzo-18-crown-6 (DNB18C6) was synthesized via the nitration reaction using a mixture of glacial acetic acid and nitric acid as the nitrating agent. Then, DAB18C6 was prepared by the catalytic hydrogenation of DNB18C6 under a hydrogen pressure of 0.35 MPa. The reaction mechanism is shown in Figure 2. The produced DAB18C6 was recrystallized twice before use.

### 2.3. Preparation of Membranes

#### 2.3.1. Preparation of Polyamide (PA) Membranes

The polysulfone membrane was cut into 13 × 13 cm pieces and soaked in a 2 g·L^−1^ sodium dodecyl sulfate solution for 48 h. After soaking, the membrane was rinsed again with pure water and air-dried for later use. m-Phenylenediamine (MPD) was dissolved in deionized water, and trimesoyl chloride (TMC) was dissolved in n-hexane. The support membrane was fixed onto a glass plate, and the MPD solution was poured over the membrane. The solution was allowed to stand to ensure the membrane surface was saturated with solute molecules. The excess aqueous solution was poured out, and the membrane was air-dried in a fume hood for a few minutes. Then, the TMC solution in n-hexane was poured onto the membrane to facilitate a rapid reaction. The entire interfacial polymerization process was conducted in a temperature- and humidity-controlled chamber, with the humidity set to 30% and the temperature set to 28 °C. After the reaction was completed, the membrane was washed with n-hexane to remove the unreacted monomers. The membrane was then washed with deionized water and heat-treated in a 60 °C oven for 11 min.

#### 2.3.2. Preparation of Crown Ether-Modified Membranes

**Figure 3:**
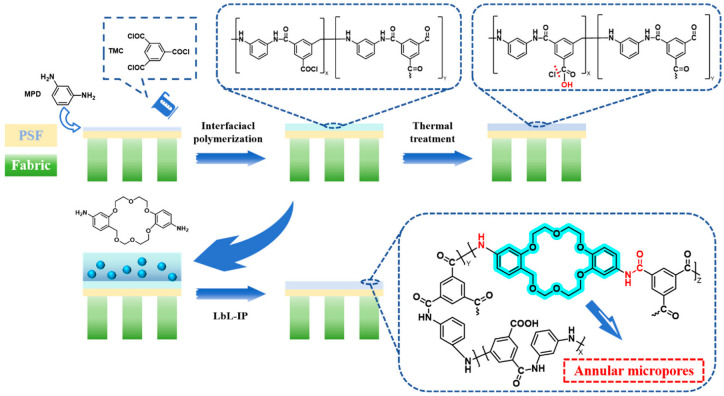
Schematic diagram of membranes preparation.

A 2 wt% aqueous solution of MPD and a 0.15 wt% n-hexane solution of TMC were prepared. The support membrane, which had been soaked in sodium dodecyl sulfate, was removed, washed thoroughly with pure water, and fixed onto a glass plate. The aqueous solution was poured onto the membrane and allowed to stand for 15 min to ensure the membrane surface was enriched with solute molecules. The membrane was placed in a fume hood for a few minutes to remove surface droplets, and then the organic phase solution was added, allowing the reaction to proceed for 60 s. Then, a 0.01 wt% methanol solution of diamino-dibenzo-18-crown-6 (DAB18C6) was poured onto the membrane (methanol was used due to the poor water solubility of the crown ethers), and the reaction was continued for another 60 s. Finally, the membrane was washed with methanol to remove the unreacted monomers and heat treated in a 60 °C oven for 11 min. The preparation process is shown in Figure 3. The polysulfone-based membrane was named M0, the unmodified MPD-TMC membrane was named M1, and the modified MPD-TMC-DAB18C6 membrane was named M2.

### 2.4. Testing and Characterization

#### 2.4.1. Characterization of Crown Ethers

The molecular structure of the prepared diamino-dibenzo-18-crown-6 and dinitrobenzo-dibenzo-18-crown-6 was determined using a nuclear magnetic resonance (NMR) spectrometer (AVANCE III HD 600 MHz, Fällanden, Switzerland), which provided the proton resonance spectrum.

The elemental composition of the compound was determined using an elemental analyzer (vario EL cube, China Teaching Equipment Instrument Co., Ltd., Beijing, China), which accurately measured the mass ratio of each element. Testing was performed in two modes: CHNS mode and O mode. Approximately 5 mg of the sample was weighed, encapsulated, and analyzed in both modes.

#### 2.4.2. Characterization of the Fabricated Membranes

A Fourier-transform infrared spectrometer (FT-IR Nicolet iS50, Thermo Fisher, Waltham, MA, USA) was used to obtain FT-IR spectra to identify specific functional groups in the membranes, analyze their structure, and assess whether interfacial polymerization was complete. Clean membrane samples prepared by two methods were tested using the attenuated total reflection (ATR) technique.

The elemental composition and content of the membrane surface were analyzed using an X-ray photoelectron spectrometer (ESCALAB Xi+, Thermo Fisher Scientific, Waltham, MA, USA). Dried samples (5 mm × 5 mm) were mounted on a sample holder using conductive adhesive. Monochromatic aluminum-Kα radiation was used as the X-ray source, with a photoelectron incidence angle of 60°. The scanning range was set from 100 to 1000 eV, with a step size of 1 eV. Surface chemical properties were evaluated to a depth of 10 nm.

A scanning electron microscope (SEM MAIA3 LMH, Brno, Czech Republic) was used for detailed microscopic analysis, particularly for observing cross-sectional characteristics, surface morphology, and microstructure. Membrane samples were torn from the non-woven fabric, frozen in liquid nitrogen for brittle fracture, and mounted with conductive adhesive. Gold sputtering was performed before imaging to address the low conductivity of the membranes. And the roughness of the membrane was characterized using an atomic force microscope (AFM SPM-9700HT, SHIMADZU, Kyoto, Japan).

The hydrophilicity of the membrane surface was detected by a contact angle goniometer (DSA100, KRUSS, Hamburg, Germany). At least five random points were measured for each sample. Membranes were cut into 1.5 × 1.5 cm pieces and adhered to clean glass slides for testing. During testing, approximately 5 µL of deionized water was dropped onto the membrane surface, and the contact angle (θ) was recorded to assess wettability.

#### 2.4.3. Separation Performance Evaluation

**Figure 4:**
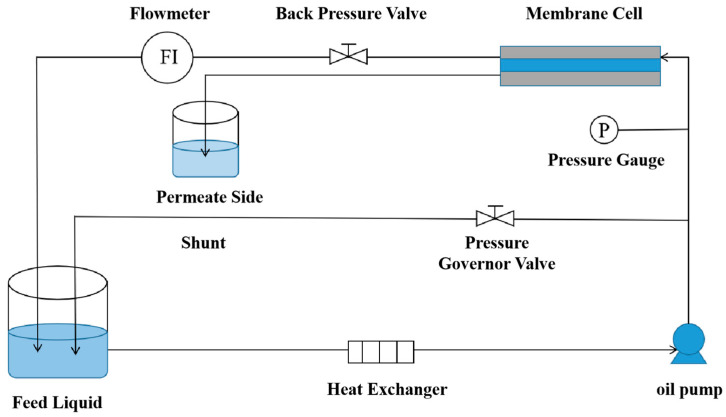
Diagram of the Testing Device.

As shown by Figure 4, a custom crossflow cell was used to evaluate membrane performance under varying feed pressures, feed concentrations, and different salt solutions. Four types solutions (Na_2_SO_4_, MgSO_4_, NaCl, and MgCl_2_) with different concentrations (1 g·L^−1^, 1.5 g·L^−1^, 2 g·L^−1^, and 2.5 g·L^−1^), respectively, were applied in this work. The crossflow system was equipped with a pressure gauge before the membrane cell and a back pressure valve after it to regulate feed pressure. The feed solution was pre-mixed by bypass operation, ensuring homogeneity. Pressure and back-pressure were adjusted to exceed 17 bar, and the system was operated for over 30 min to compact the membrane. Permeate was collected, and its volume and conductivity were measured. Membrane permeability and salt rejection were calculated using Equations (1) and (3). Each test was repeated at least five times to obtain an average.

Pure Water Permeability (*F*):

(1) $$F=\frac{V}{A\cdot t\cdot P}$$

where *F* is pure water permeability (L·m^−2^·h^−1^·bar^−1^), t is test duration (h), *V* is the permeate volume (L), P is the operating pressure (bar), and *A* is the effective membrane area (m^2^).

Salt Rejection (*R*):

(2) $$R=\left(1-\frac{C_{p}}{C_{f}}\right)\times 100\%$$

where *C_p_* is the concentration on the permeate side (g·L^−1^), *R* is the salt rejection (%), and *C_f_* is the concentration on the feed side (g·L^−1^). When the concentration of the feed liquid is very low, due to the approximately linear relationship between conductivity and ion concentration, we can substitute conductivity for ion concentration to calculate the desalination rate [26]. Substitute $C_{f}=\frac{\sigma_{f}}{k}$ and $C_{p}=\frac{\sigma_{p}}{k}$ into the original desalination rate formula. So the salt rejection can be approximately calculated as follows:

(3) $$R=\left(1-\frac{\sigma_{p}}{\sigma_{f}}\right)\times 100\%$$

where *R* is the salt rejection (%), *σ_p_* is the permeate conductivity (µS·cm^−1^), and *σ_f_* is the feed conductivity (µS·cm^−1^).

## 3. Results and Discussion

### 3.1. Characterization Results of Crown Ethers

**Figure 5:**
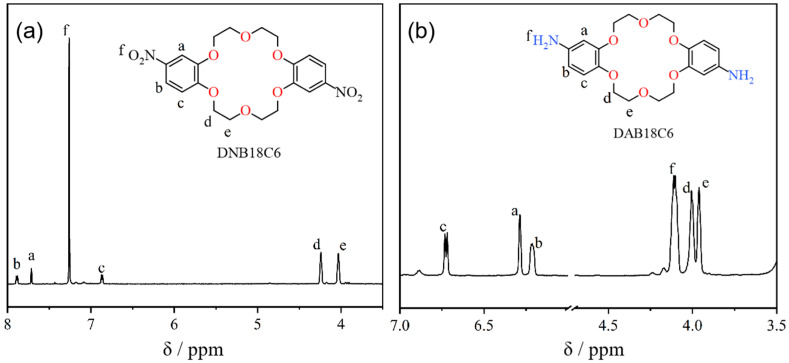
Nuclear magnetic resonance spectra of crown ethers. (**a**) Nuclear magnetic resonance spectra of DNB18C6. (**b**) Nuclear magnetic resonance spectra of DAB18C6.

The molecular structure of the crown ether was precisely characterized using nuclear magnetic resonance (NMR) spectroscopy. As shown in Figure 5a, the characteristic peaks observed between 6.82 and 7.94 ppm are attributed to the hydrogen atoms on the benzene rings [25]. The hydrogens at symmetrical positions on both benzene rings (a, b, c) are equivalent, with two hydrogens at each position, indicating that a single hydrogen atom on each benzene ring has been substituted. The peaks labeled d and e correspond to the methylene hydrogens in the crown ether’s macrocyclic ring, which appear as multiplets. Figure 5b presents the NMR hydrogen spectrum of DAB18C6. The peaks labeled a, b, and c correspond to the characteristic hydrogens on the benzene rings. Compared to the spectrum of DNB18C6, these peaks exhibit a shift toward a higher field, attributed to the shielding effect caused by the electron-donating -NH_2_ group.

Additionally, an elemental analyzer was employed to accurately determine the mass ratios of the elements in the compound, providing insights into the sample’s elemental composition and molecular formula. Elemental analysis was performed in two modes: CHNS and O. Approximately 5 mg of the sample was prepared and tested in both modes. As shown in Table 1, the elemental analysis results indicate that the actual carbon-to-nitrogen ratio aligns well with the theoretical value. However, the carbon-to-hydrogen ratio is slightly lower than expected, while the oxygen content is marginally higher. These minor discrepancies may be attributed to incomplete drying of the DNB18C6 sample prior to testing. Table 2 shows that the elemental ratios for DAB18C6 closely match the theoretical values, confirming the successful synthesis of a relatively pure amino crown ether product through hydrazine reduction.

### 3.2. Chemical Composition of Membrane Surface

**Figure 6:**
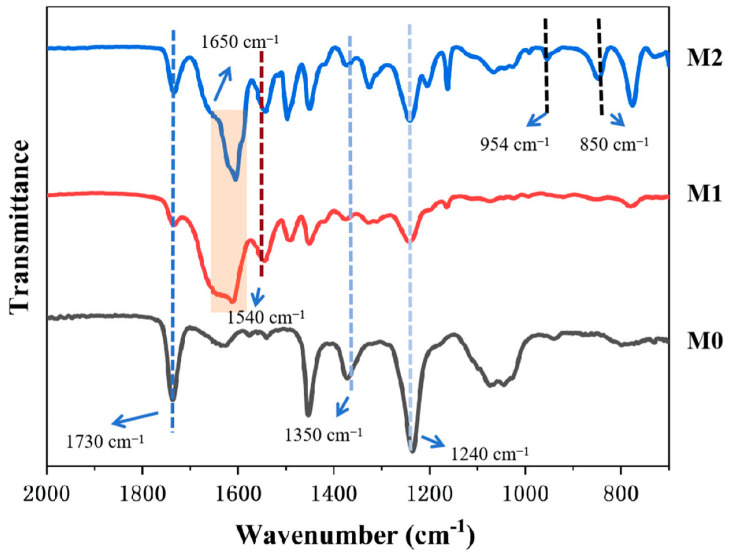
ATR-FTIR spectra of membrane M0 (base membrane), membrane M1 (PA membrane), and membrane M2 (diamino crown ether-modified membrane).

**Figure 7:**
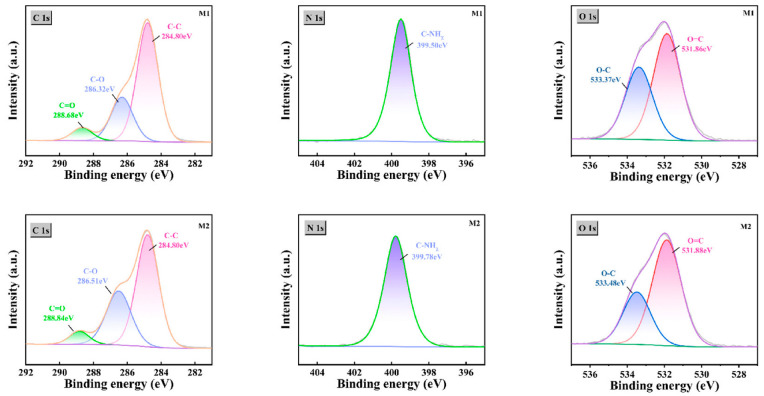
XPS spectra of C 1s, N 1s, and O 1s for M1 and M2.

In this study, the prepared crown ethers, m-phenylenediamine (MPD) and trimesoyl chloride (TMC) were used to perform secondary interfacial polymerization on a polysulfone-based membrane to synthesize the samples. To investigate the specific chemical composition of the membrane surface, attenuated total reflectance Fourier-transform infrared spectroscopy (ATR-FTIR) was employed to characterize the chemical groups and components, thereby verifying the reaction. As shown in Figure 6, absorption peaks at 1730 cm^−1^ and 1240 cm^−1^ correspond to the characteristic infrared peaks of ester groups (-COO-) [27]. These peaks are attributed to the polyester nonwoven substrate present in the polysulfone-based membrane. The peak at 1350 cm^−1^ represents the stretching vibration of sulfone groups [28]. Furthermore, all MPD/TMC-based polyamide membranes exhibit characteristic absorption peaks at 1650 cm^−1^ and 1540 cm^−1^. This indicates that the interfacial polymerization was successful, and the desired polyamide (PA) membrane was successfully polymerized onto the base membrane. These peaks correspond to the stretching vibration of the carbonyl group in amide bonds and the in-plane bending vibration of the N-H bond in amide bonds, respectively [29,30]. Additionally, for crown ether-modified membranes, significantly enhanced peaks are observed at 954 cm^−1^ and 850 cm^−1^, which correspond to the characteristic ether bond peaks introduced by the crown ether modification [25]. This group was not detected in the other two membranes, indicating the successful introduction of the crown ether. Furthermore, X-ray photoelectron spectroscopy (XPS) was used for an in-depth analysis of the membrane surface groups. As shown in Figure 7, for the unmodified membrane (M1), the fitted oxygen (1s) peaks are observed at 533.4 eV and 531.9 eV, corresponding to C-O (single bond) and C=O (double bond), respectively. Notably, for the crown ether-modified membrane (M2), the oxygen (1s) fitted peak corresponding to C-O (single bond) shows a significant shift, confirming the successful incorporation of crown ether on the modified membrane.

### 3.3. Membrane Morphology

**Figure 8:**
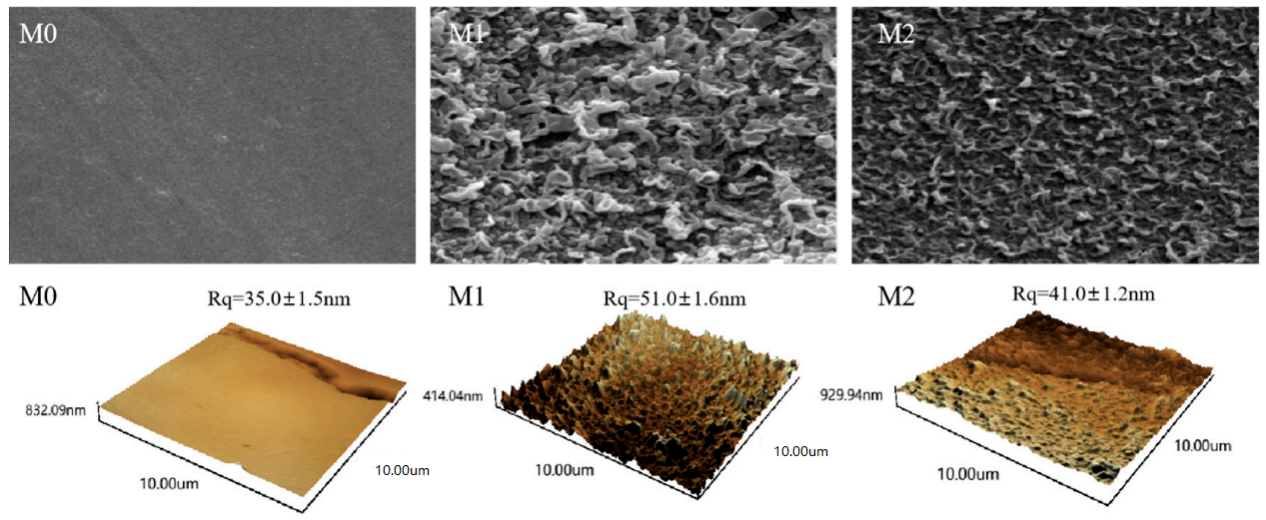
The surface images of membranes evaluated by SEM and AFM.

Figure 8 presents electron microscopy images of the polysulfone support membrane, the PA membrane, and the crown ether-modified PA membrane. The polysulfone support membrane exhibits a smooth, clean surface and a porous cross-section. While, the membrane after the IP reaction showed a nodular, uneven surface structure. This unevenness arises from the poor interfacial stability during the reaction, primarily due to the MPD concentration gradient at the interface. Comparing M2 with M1 in Figure 8 reveals that incorporating a crown ether into the interfacial polymerization process produces a denser and more uniform membrane surface. This indicates that the amino-functionalized crown ether effectively reacts with the residual acyl chloride monomers after the initial interfacial polymerization. Consequently, the incorporation of the crown ether unavoidably increases the cross-sectional thickness of the membrane, which now reaches the micron scale. As shown in the atomic force microscopy (AFM) images shown in Figure 8, the surface roughness of the modified membrane decreases from 51.0 ± 1.6 nm for M1 to 41.0 ± 1.2 nm for M2. This finding aligns with the earlier SEM analysis, confirming that crown ether was successfully incorporated onto the PA membrane surface through interfacial polymerization, slightly reducing the surface roughness of the reverse osmosis (RO) membrane [31,32]. This phenomenon indicates that the crown ether-modified membrane’s smoother surface helps reduce the accumulation of contaminants, thereby enhancing its antifouling performance, which has been verified by Goh et al. [33].

### 3.4. Surface Hydrophilicity of Membranes

**Figure 9:**
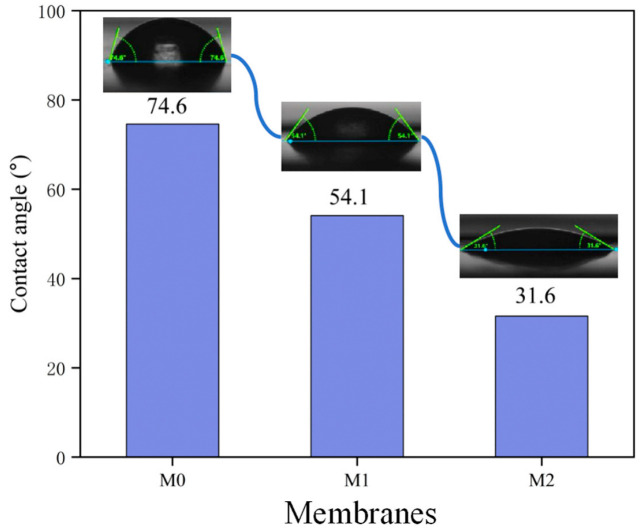
Water contact angle of membranes M0, M1, and M2.

It is well known that the surface hydrophilicity of reverse osmosis (RO) membranes significantly affects their permeability, rejection performance, and antifouling properties [34]. Therefore, the water contact angle (WCA) of the fabricated RO membranes was investigated. As shown in Figure 9, the water contact angle decreased from 74.6° for the support membrane to 54.1° for the PA membrane and further decreased to 31.6° for the crown ether-modified membrane. This result indicates that the introduction of crown ether groups significantly enhanced the hydrophilicity of the modified membrane. A strong hydration layer could be formed near the surface of hydrophilic membranes, which facilitated water molecule transport and thereby increased water permeability. Moreover, the hydrophilic surfaces exhibit a lower fouling tendency compared to those with hydrophobic surfaces [35,36,37].

### 3.5. Perm-Selectivity Evaluation

**Figure 10:**
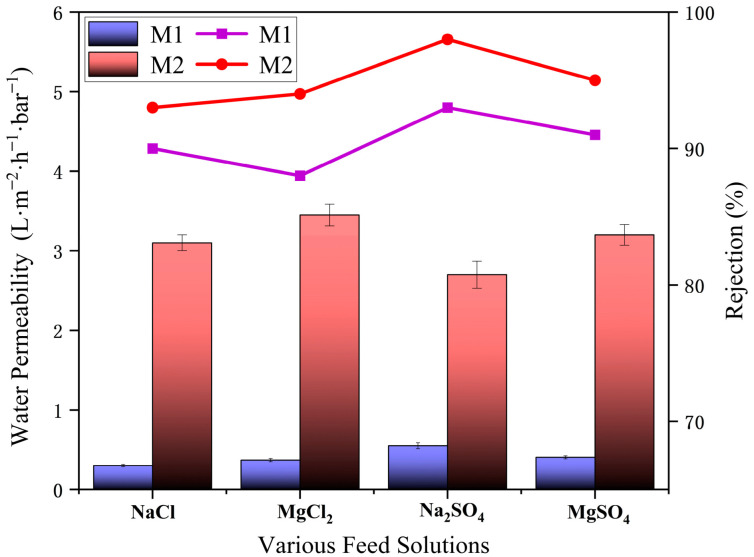
Performance with different salt solutions.

At 25 °C, the membrane’s performance was evaluated using a cross-flow setup under various feed pressures, feed concentrations, and salt solutions. Four different salt solutions with the same concentration (2 g·L^−1^) were applied to assess the membrane’s desalination performance. As shown in Figure 10, significant improvement in both permeability and salt rejection was observed for the crown ether-modified PA membrane. The permeability of crown ether-modified was 4.4–10.5 times higher than unmodified PA membrane for different salt solutions. Also, the salt rejection showed an obvious improvement. For example, the crown ether-modified membrane exhibited permeability of 3.12 L·m^−2^·h^−1^·bar^−1^ with a salt rejection of 93.2% for 2 g·L^−1^ NaCl solution, while the permeability unmodified membrane was only 0.27 L·m^−2^·h^−1^·bar^−1^ with salt rejection of 89.3%. Therefore, the incorporation of crown ether can facilitate the permeation of water and reject salt. Water molecules are smaller than the micropore of crown ether. So, water can easily permeate through the membrane. At the same time, the micropore can reject the larger ion hydrate, which can improve the salt rejection. This phenomenon indicated that we successfully built a water rapid transport channel in the membrane. Interestingly, the rejection order for various salt solutions was ranked as follows: Na_2_SO_4_ > MgSO_4_ > NaCl > MgCl_2_ for both the unmodified PA membrane and the crown ether-modified PA membrane. This phenomenon can be attributed to the membrane’s surface charge and pore size. The micropores grafted with crown ether have diameters smaller than the ionic radii, while hydrolysis of acyl chloride groups during thermal treatment produces carboxylate groups (-COO-), imparting a negative charge to the membrane surface. This negative charge strongly repels anions, enhancing rejection.

The effects of operating pressures on membrane were investigated using a 2 g·L^−1^.

**Figure 11:**
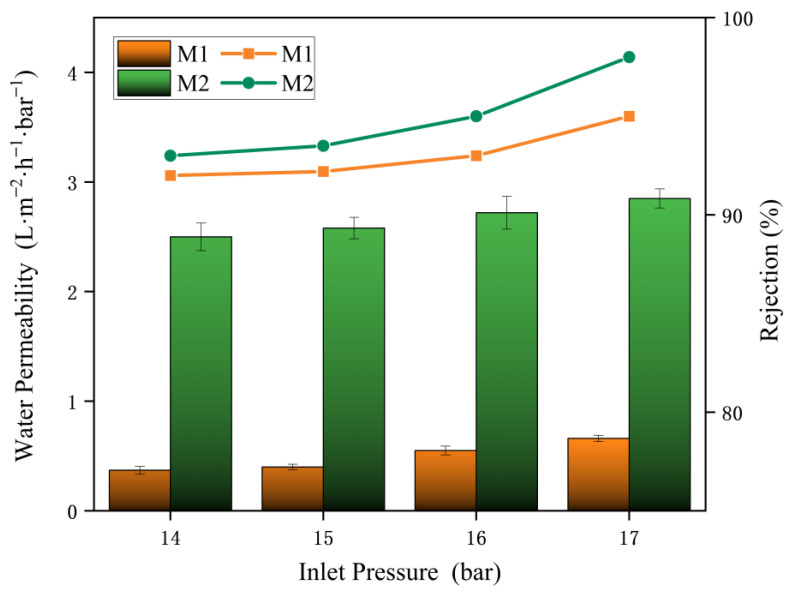
Performance under varying pressures.

Na_2_SO_4_ solution, and the results was shown in Figure 11, and the results were shown in Figure 11. The permeability of two kinds of membrane increased with the pressure. This can be explained by the fundamental principles of reverse osmosis, where a pressure gradient drives the separation process. The permeability of the unmodified PA membrane (M1) increased from 2.52 L·m^−2^·h^−1^·bar^−1^ to 2.75 L·m^−2^·h^−1^·bar^−1^, while the permeability of the diamine crown ether-modified membrane (M2) rose from 0.38 L·m^−2^·h^−1^·bar^−1^ to 0.62 L·m^−2^·h^−1^·bar^−1^, showing a significant improvement. Increasing the external pressure inevitably enhances the permeability [38]. This improvement is attributed to the screening effect of the crown ether on water molecules at the membrane surface. The crown ether-modified PA membrane (M2) consistently exhibited higher rejection rates than the unmodified PA membrane (M1). Both membranes showed an increasing trend in the rejection rate with rising pressure. Notably, the permeability and rejection rate of the modified membrane were superior, with rejection exceeding 99% at 17 bar.

**Figure 12:**
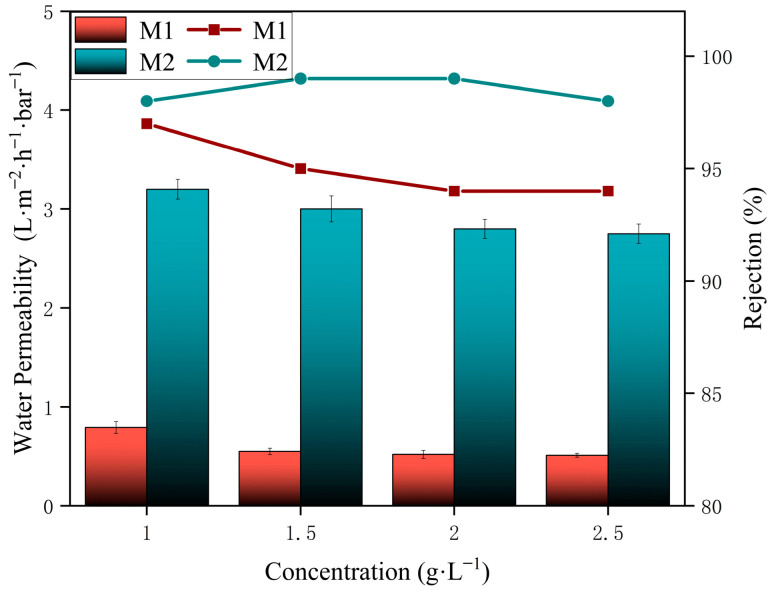
Performance with different concentrations.

The effects of feed salt concentrations on the permeability and rejection rate were also studied. At lower salt concentrations, water permeability is higher. As the salt concentration increases, the osmotic pressure difference across the membrane rises, reducing the driving force for solvent transport and consequently lowering the permeability. As shown in Figure 12, the permeability of both M1 and M2 decreased with higher salt concentrations. In summary, the PA membrane modified through secondary interfacial polymerization with crown ether demonstrated significant enhancements in both permeability and rejection rates compared to the unmodified PA membrane. This improvement can be attributed to the increased hydrophilicity imparted by crown ether, which forms a hydration layer on the membrane surface, thereby boosting permeability. Additionally, the microporous structure of the crown ether effectively sieves water molecules at the membrane surface, achieving rejection rates exceeding 99%.

**Figure 13:**
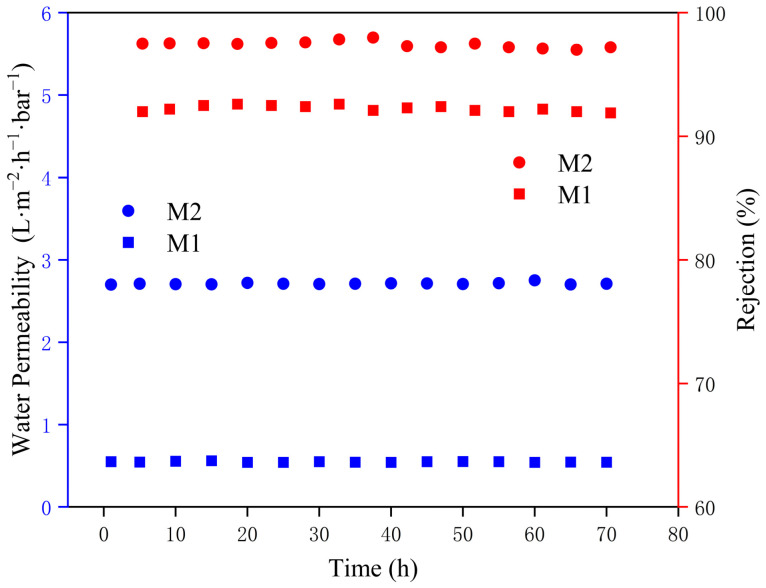
Stability performance test.

In reverse osmosis applications, the separation performance is a key metric for evaluating membrane efficiency, but long-term stability is equally important. Greater stability reduces replacement frequency, effectively lowering costs, minimizing manual intervention, and decreasing wear on equipment. Figure 13 presents the long-term operational stability of the fabricated PA membrane (M1) and the modified membrane (M2), tested under 17 bar using a 2 g·L^−1^ Na_2_SO_4_ solution, with continuous replenishment of the feed solution to maintain stable concentration. The modified membrane maintained a stable separation performance after 72 h of testing, with a permeability of 2.8 L·m^−2^·h^−1^·bar^−1^ and a rejection rate exceeding 99%. The permeability of the unmodified membrane is maintained at 0.5 L·m^−2^·h^−1^·bar^−1^, with a retention rate above 92%. In contrast, the modified membrane M2 showed notable improvements in both permeability and selectivity. Both permeability and rejection were higher than those of the unmodified PA membrane.

In modern reverse osmosis seawater desalination technology, selective barrier layers must achieve salt rejection rates consistently above 98.5% [39]. The results demonstrate that the sulfonamide-grafted membrane in this study exhibited enhanced water permeability and excellent stability during long-term operation.

## 4. Conclusions

Crown ether-modified RO membrane was developed using the layer-by-layer interfacial polymerization (LbL-IP) method. The first PA layer was prepared following the traditional IP method. The amino group functioned dibenzo-18-crown-6 (DAB18C6) was synthesized by the nitration and hydrogenation reaction of dibenzo-18-crown-6 (DB18C6). After interfacial polymerization between m-phenylenediamine (MPD) and trimesoyl chloride (TMC), DAB18C6 solution in methanol was poured on the membrane to react with the residual TMC. The pore sizes of the 18-crown-6 were larger than water molecules but smaller than hydrated ions. At the same time, the ether groups in crown ether displayed good hydrophilicity. Their size-selective properties and affinity for water molecules enable high permeability and high rejection rates.

The results showed that the introduction of the crown ether groups significantly enhanced the hydrophilicity of the membrane surface, as evidenced by reduced water contact angle (WCA) from 74.6° for the support membrane to 54.1° for the PA membrane and further to 31.6° for the modified membrane. The crown ether-modified membrane possessed both higher permeability and higher salt rejection due to the size-selective micropore and the good hydrophilicity. This study demonstrated the significant enhancement in permeability and rejection rates of the modified membranes under varying feed pressures, concentrations, and different salt solutions. Overall, this modification strategy offers a promising approach and potential pathway for the future development of reverse osmosis membranes.

## Figures and Tables

**Table 1:** Elemental Analysis Results of DNB18C6.

	C/N	C/H	N/H	Oxygen Content
Theoretical ratio	8.57	10.91	1.27	37.9%
Actual ratio	8.57	10.84	1.26	38%

**Table 2:** Elemental Analysis Results of DAB18C6.

	C/N	C/H	N/H	Oxygen Content
Theoretical ratio	8.57	9.23	1.08	26.5%
Actual ratio	8.57	9.22	1.08	26.5%

## Data Availability

Data are contained within the article.

## Abbreviations

The following abbreviations are used in this manuscript:

AFM	Atomic force microscopy
DAB18C6	Diamino-dibenzo-18-crown-6
DNB18C6	Dinitrobenzo-dibenzo-18-crown-6
FT-IR	Fourier-transform infrared spectrometer
IP	Interfacial polymerization
LbL-IP	Layer-by-layer interfacial polymerization
MPD	M-phenylenediamine
NMR	Nuclear magnetic resonance
NF	Nanofiltration
PA	Polyamide
RO	Reverse osmosis
SEM	Scanning electron microscopy
TFC	Thin-film composite
TMC	Trimesoyl chloride
WCA	Water contact angle
XPS	X-ray photoelectron spectroscopy
